# Social Identities as Pathways into and out of Addiction

**DOI:** 10.3389/fpsyg.2015.01795

**Published:** 2015-11-30

**Authors:** Genevieve A. Dingle, Tegan Cruwys, Daniel Frings

**Affiliations:** ^1^School of Psychology, The University of Queensland, St Lucia, QLD, Australia; ^2^Centre for Youth Substance Abuse Research, The University of Queensland, Brisbane, QLD, Australia; ^3^Department of Psychology, London South Bank University, London, UK

**Keywords:** social identity, social support, substance misuse, treatment, thematic analysis

## Abstract

There exists a predominant identity loss and “redemption” narrative in the addiction literature describing how individuals move from a “substance user” identity to a “recovery” identity. However, other identity related pathways influencing onset, treatment seeking and recovery may exist, and the process through which social identities unrelated to substance use change over time is not well understood. This study was designed to provide a richer understanding of such social identities processes. Semi-structured interviews were conducted with 21 adults residing in a drug and alcohol therapeutic community (TC) and thematic analysis revealed two distinct identity-related pathways leading into and out of addiction. Some individuals experienced a loss of valued identities during addiction onset that were later renewed during recovery (consistent with the existing redemption narrative). However, a distinct identity gain pathway emerged for socially isolated individuals, who described the onset of their addiction in terms of a new valued social identity. Almost all participants described their TC experience in terms of belonging to a recovery community. Participants on the identity loss pathway aimed to renew their pre-addiction identities after treatment while those on the identity gain pathway aimed to build aspirational new identities involving study, work, or family roles. These findings help to explain how social factors are implicated in the course of addiction, and may act as either motivations for or barriers to recovery. The qualitative analysis yielded a testable model for future research in other samples and settings.

‘I don’t ever wanna drink again…I just need a friend.’

*—Amy Winehouse, lyrics from “Rehab”*

## Introduction

Although theories of addiction have historically focused on individual factors (such as biological, cognitive behavioral, and individual differences models), a growing body of research shows that social factors also play an important role. Social factors are involved at every stage of the development of and recovery from a substance use disorder (SUD). For example, the onset of problematic substance use may be associated with social isolation (Chou et al., 2011) or with peer pressure and normative influences on behavior (Bauman and Ennett, 1996; Ary et al., 1999). For individuals in addiction treatment, social network support for abstinence influences treatment outcomes (Zywiak et al., 2009; Kelly et al., 2011; Litt et al., 2015). Furthermore, two of the three most common reasons for relapse into substance misuse after treatment are social: namely, interpersonal conflict and social pressure to use (Hodgins et al., 1995; Zywiak et al., 2006). In this paper, we begin by summarizing existing research about the influence of social factors on the trajectory of substance use. We then extend this work with a qualitative analysis of interviews conducted with adults in a drug and alcohol therapeutic community (TC) to begin to determine if, and how, their social relationships and social identities play a role in the pathways into and out of addiction.

### Social Factors Involved in the Onset and Development of Addiction

#### Peer Normative Influence

A variety of factors have been implicated in the onset of substance use in adolescents. Although family factors play a role, peer influence (both indirectly through modeling substance use, and directly through provision of substances and encouragement to use) is widely considered to be the most consistent and influential factor (Newcomb and Bentler, 1989). For instance, Ary et al. (1999) conducted a 24-month longitudinal study of 204 adolescents and parents. They found that a composite measure of problem behavior that included substance use at a 2-year follow up was directly predicted by associations with deviant peers, and indirectly influenced by poor parental monitoring in a pathway mediated by associations with deviant peers.

Amongst university students, several norm-based prevention programs have been based on the provision of information about descriptive norms (e.g., telling students about the quantity and frequency of drinking of other university students), and injunctive norms (e.g., telling students that they ought to stay under the safe drinking guidelines). The results of such programs have been mixed, and recent research indicates that the effectiveness of such information depends on whether the individuals consider themselves to be prototypical of the group or unique (Goode et al., 2014).

#### Social Isolation and the Need to Belong

Social isolation (that is, a lack of peer connections) has also been related to risk of substance misuse. In the case of smoking for example, Ennett and Baumann (1993) conducted a social network analysis of 1,092 adolescents in five junior high schools and placed them into three categories: cliques (several members of a group named other members as their best friends); liaisons (who had at least two links with either clique members or other liaisons), or isolates (who had few or no connections to other adolescents in their school). They found that across all schools, isolates were far more likely to be current smokers than either clique members or liaisons. The percentage of clique members who were smokers ranged from 3.9 to 15.5%, compared with 6.7% up to 38.5% of isolated adolescents across schools.

Despite the potential negative consequences of “peer pressure,” social relationships bring a wide range of tangible and psychological relationships. It has been argued that social connectedness provides a sense of belonging, purpose, and meaning that are key psychological needs (Baumeister and Leary, 1995) and critical to mental health (Cruwys et al., 2014a). In light of this, it is interesting to note that population studies have suggested that SUD is associated with very high levels of social isolation (Chou et al., 2011). A recent investigation of people in TC treatment for SUDs found that 63% were single; 69% not in fulltime employment; 24% had “0 or 1” close friend; and 42% spent most of their time “alone” (Dingle, 2012). Less research has explored the causal direction of this effect. Some evidence points to social isolation as an outcome of substance use (e.g., Link et al., 1997; Vangeest and Johnson, 2002), whereas other studies suggest social isolation has a causal role in the development of SUD (e.g., Bellis et al., 2012; Wakefield et al., 2015). It is possible that both pathways may be at work, but apply to different sub-populations of individuals with SUDs. Equally, such effects could be reciprocal.

In summary, one extant body of research shows that family, peer, and social groups can influence (protect against or promote) the onset and development of problematic substance use. A second body of research suggests that socially isolated and marginalized people may be at increased risk of substance use. Theoretically, substance use may be a pathway to a valued social group and identity as part of a “using” social group may engender a sense of belonging. However, to date there is insufficient evidence of this second pathway and its addiction prevention and treatment implications in the available research. In the next section we consider the social factors implicated in addiction and spontaneous or assisted recovery from addiction.

### Social Factors Involved in Recovery From Addiction

In research about spontaneous (unassisted) recovery, Waldorf and Biernacki (1981) interviewed former opiate addicts and found that their decision to stop taking drugs came about when the user’s addict identity conflicted with, and caused problems for, their other social identities (such as those of partner, parent, or employee) in ways that had become intolerable. These researchers referred to recovery as a process by which individuals learn to manage a “spoiled identity”; through restoring a more positive prior identity and/or the establishment of a new one. This notion was further supported in a Scottish interview study with 70 people recovering from opiate addiction (McIntosh and McKeganey, 2000). One common theme arising from these interviews was participants’ description of a discrepancy between the person they had become while addicted and their “real” self, and a belief that recovery would mean giving expression to their true self through a rejection of their drug using past and the establishment of a new abstinent way of life. The term “spoiled identity” was also used by Hughes (2007) to describe the deviant activities (e.g., carrying a gun, theft, etc.) that opiate users engaged in order to obtain heroin.

Other research on recovery has focused on social factors involved in mutual support groups such as Alcoholics Anonymous (AA) and Narcotics Anonymous (NA). Moos (2007) identified the following ingredients of mutual support groups that were associated with long-term abstinence from alcohol and other drugs: bonding and support; obtaining an abstinence-focused role model; and doing service work within the group (Moos, 2007). These findings were echoed in a review of 24 studies focusing on how AA membership benefits people with alcohol dependence (Groh et al., 2008). Additional evidence about the mechanisms of action of AA membership comes from an analysis of data from 1,726 adults in the Project Match study (Project MATCH Research Group, 1997), which showed that adaptive social network changes and increases in social abstinence self-efficacy were the mechanisms that exerted the most influence in recovery (Kelly et al., 2011; Stout et al., 2012). A narrative analysis of life stories with 51 respondents in recovery from a range of addictive behaviors revealed five different story types which were labeled: the AA story, the growth story, the co-dependence story, the love story and the mastery story (Hanninen and Koski-Jannes, 1999). Of these, the “AA story” was consistent with the notion of social identity loss following by finding ones place within the (AA or recovery) community (i.e., a recovery and redemption narrative). In contrast, the “love story” narrative matched most closely themes of unmet identity needs and social isolation being compensated for by the addiction. For these individuals, receiving real loving care was the key to their recovery from the addiction. Other work supports the notion that building sober social networks is particularly important in light of the findings that social bonds to recovery networks are stronger, and the quality of friendships better, in non-using than in substance using networks (Humphreys et al., 2004). Furthermore, sober networks that include occupational roles and peers have been found to support and destigmatize the recovery process of individual members (Best, 2015). By contrast, maintaining ties with using networks is associated with relapse and poorer outcomes following treatment (Havassy et al., 1991; Dingle et al., 2015).

In summary, the prevailing view suggests that people with SUDs lose important social identities with the onset of addiction and they are motivated to restore these lost identities during recovery. What is not known is whether alternative identity related pathways exist during the course of addiction. Also lacking in this growing body of social addiction research is a unifying theory of social relationships. In the next section we review emerging evidence for one such theory—the social identity approach (Tajfel and Turner, 1979; Turner et al., 1987)—applied in the domain of substance misuse.

### A Social Identity Approach to Addiction

A central tenet of the social identity approach is that our social group memberships inform our self-concept—that is, “who *I* am” is defined, at times and in part, by “who *we* are” (Tajfel and Turner, 1979; Turner et al., 1987). This process of *identification* with social groups is a foundation of social behavior, such that when we identify with a social group, this influences how we perceive the world and what we choose to do, including in the domain of health (Platow et al., 2007; Haslam et al., 2009; Cruwys et al., 2012). Furthermore, social identity makes social support possible, as people are more able to give and receive support from members of groups that they identify with (Haslam et al., 2005). It follows therefore, that defining oneself as a “drinker,” “stoner,” or “junkie” has enormous implications for substance use behavior (Schofield et al., 2001). This is of particular importance for people who are socially isolated, as their main (or only) source of social support and self-definition is likely to be their substance-using social groups, and their behaviors may thus be guided to a significant extent by “addict” related identities. Conversely, thinking of oneself as belonging to a *recovery* group or network may influence a person to persist with abstinence or distance themselves from substance-using networks (Best et al., 2014).

Using this theoretical perspective, Frings and Albery’s, (2015) Social Identity Model of Cessation Maintenance argues that social identities built around cessation can support successful outcomes through a combination of increased self-efficacy, self-esteem and an adjustment of which behavioral norms are acted upon, bolstered by both social support and social control enacted by other group members. In support of this approach, Buckingham et al. (2013) examined the role identity played in maintaining abstinence amongst members of mutual support groups (AA and NA). They found that participants who evaluated their recovery identity more favorably than their addiction identity (termed “evaluative differentiation”) had significantly lower relapse rates and reduced appetitive behaviors. A stronger endorsement of the recovery identity relative to the addiction identity (“identity preference”) was related to higher levels of abstinence self-efficacy, which predicted number of months drug-free and reduced levels of appetitive behaviors. Identity preference was also related to higher self-efficacy, which in turn was related to lower relapse.

Beckwith et al. (2015) investigated factors related to client identification with a TC in the first 2 weeks. Those who experienced an increased identification with the TC and reduced social identification with their substance using groups over the first 2 weeks, stayed in treatment significantly longer than the others (Beckwith et al., 2015). A prospective longitudinal study of 132 adults from this same TC (Dingle et al., 2015) reported that for most participants, identification as a substance user continued to decrease among those who stayed in treatment, with 76% of the sample reporting a decrease in user identity strength over the first month in the TC. At the same time, recovery identification increased significantly over time, with 64% of the sample staying the same or increasing their recovery identity ratings over the first month. The increase in recovery versus user identification over time accounted for 33–50% of the variance in drinking and wellbeing at follow up (Dingle et al., 2015).

### The Current Study

A growing body of research suggests that the change from an addiction/user identity to a recovery identity is a critical ingredient in successful treatment. This existing research emphasizes an identity loss and renewal or “redemption” narrative associated with the onset and recovery from addiction. However, it is unclear at this stage whether this narrative fits all people’s experience or whether other identity related pathways exist in the addiction context. Nor does such a narrative account explore adequately how identity concerns facilitate addiction onset. A final area of research that is currently underdeveloped is a description of the range of other (non-substance related) social identities (such as family, occupational, recreational, religious) that exist among people with an addiction and how these broader social identities might play a role in addictive and recovery. We explored these issues through the use of qualitative methods allowing for a rich and detailed analysis of individuals’ experience. Qualitative methods have not been widely used in research on the social identity approach to health so this study is also novel in its application of this methodology to these phenomena. The study was designed to address the following specific questions:

1. What social identities and relationships do participants hold prior to their addiction?
2. What social factors are related to the onset and development of an addiction problem, and the initiation of treatment?
3. How do participants’ social identities and relationships change during treatment in a therapeutic community?
4. What social identity and relationships do participants aspire to hold after leaving treatment?

## Materials and Methods

### Participants

Twenty-one people volunteered to be interviewed for the study which was conducted as part of a larger quantitative study. Efforts were made to ensure that the sample for this study were representative of the TC as a whole. The sample comprised 69% males, aged 26–58 years (mean age = 35.9, SD = 8.9 years). A majority of participants was single (58%), a third (33%) were separated from their partner, and 8% were in a relationship. Most did not have children residing with them (85%), and the remainder had between one and four dependent children. The average number of close friends was 4.08 (SD = 2.68). The average years of education completed was 10.77 (SD = 2.09 years). Most were not in full time work at the time they entered the TC, and the average days of paid work in the month prior to treatment was 6.62 (SD = 11.21 days). Most clients at the TC had been using more than one substance, although they nominated their primary substance of concern as alcohol (31%), heroin (15%), other opiates (8%), amphetamines (15%), and cannabis (15%). The remainder (15%) reported poly-substance use as their primary concern. The average number of years using the primary substance was 12.99 (SD = 5.98 years). Clients had completed three previous drug or alcohol treatments on average (SD = 2.18).

### Measures and Interview

#### Addiction Severity Index (McLellan et al., 2006)

The 5th edition of this semi-structured clinician-administered interview was used to assess client status in seven functional domains: alcohol and drug use, medical and psychiatric health, employment/financial support, family relations, and illegal activity. The ASI-5 is the most widely used structured interview for substance abuse and related problems, and it has adequate-to-good psychometric properties in English and a range of other languages (Snow and Tipton, 2009). Lifetime and past 30 days incidence and severity data are collected for each aspect of these domains. For the current study, participants’ demographics and substance use variables were taken from this measure.

#### Semi-Structured Qualitative Interview

The interview was designed to obtain detailed client experiences without leading them onto any particular topic. In particular, the questions contained no cues to social relationships or social identity as a topic, in an effort to only capture spontaneously generated identity related themes. Three open questions were used in each interview:

1. What was your life like before you came into [*name of the therapeutic community*]?
2. What has been your experience here at [*name of the therapeutic community*]?
3. What have you learned at [*name of the therapeutic community*] that you think will help you when you leave?

### Procedure

This study was carried out in accordance with the recommendations of The University of Queensland research ethics committee (approval #2011000953) and the London South Bank University Research Ethics Committee (approval #UREC1444) with written informed consent from all subjects. All subjects gave written informed consent in accordance with the Declaration of Helsinki. The first author and two clinically trained research assistants addressed the whole community to tell them about the broader project and to invite residents to participate in the interviews. Residents were assured that their participation was voluntary and would not affect their treatment in any way. Consenting participants were interviewed at a time that was suitable for them, in a quiet place within the TC. The interviews were audio recorded and transcribed verbatim. A thematic analysis was conducted by the three authors (two of whom were independent of the data collection process) to establish inter-rater reliability. We followed the thematic analysis procedure described by Braun and Clarke (2006) in which transcripts are read and coded independently by the coders to draw out primary themes and subthemes. The three coders then met to discuss and refine the themes on two occasions until there was a consensus.

## Results

**FIGURE 1:**
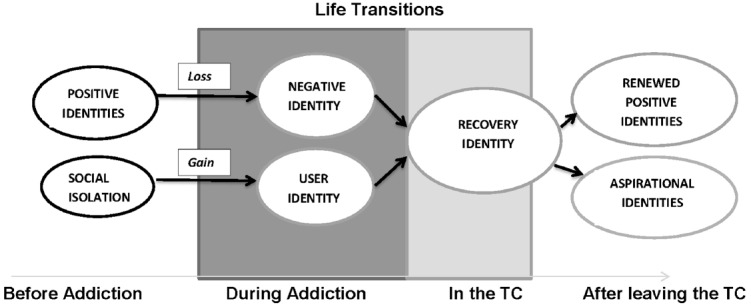
**Thematic analysis of interviews with clients in therapeutic drug and alcohol treatment showing changes in social identities over time**.

Two main identity-related “pathways” into addiction emerged in participants’ descriptions their life experiences before they became addicted. In the first pathway, participants held positive social identities prior to addiction and felt that these were lost as a result of, or alongside of, their increasing engagement in substance use and the activities related to obtaining substances. This resulted in the development of an identity which was stigmatized due to drug use, or one “spoiled” by criminal activity. In contrast, in the second pathway, participants described negative early life experiences and profound social isolation due to a lack of positive social connections and identities. For this group, the development of an addiction brought with it a new valued identity as a substance user, along with a sense of belonging and acceptance within a substance using social network. The major themes are represented in the thematic map in Figure 1. We describe the major themes along with quotations from the interviews below.

### First Pathway: Loss of Positive Identities

Many participants described a range of positive social relationships and identities before the onset of their addiction, and also spoke of a loss of identities and connections associated with addiction. For example, some described themselves as happy children, while others emphasized their achievements at school or sports:

In my good bits, I excel, really excel. I was playing basketball too, got drafted for the Northern Tigers in the under 16s, I was like 15 and competing for the Olympics, I had a really beautiful girlfriend when I was 16. And then everything just went *poof*—Male, 1 week in treatment for amphetamines.

Several participants valued their identity as a “good parent”:

*When I had my daughter I was a really good Mum for two years…no problem. I had had drug problems before but when I had her I just stopped, I was happy being a Mummy*—Female, 1 week in treatment for amphetamines.

These participants clearly saw their substance use and the deviant activities related to substance use (such as crime and deceiving family members) as problematic, and associated their using with a stigmatized label such as “junkie” and “dealer,” or seeing themselves as part of a “wrong crowd”:

*Yeah, so then I started using needles about 3 years into the relationship. Got caught up in the wrong crowd and started doing a lot of crime, a lot of bad stuff. Doing people over. I cheated on [my girlfriend] a heck of a lot*—Male, 2 weeks in treatment for heroin.

### Second Pathway: Social Isolation and Gain of Identity with Addiction

The second identity related pathway into addiction tended to involve a lack of social relationships and supports. Sometimes the experience of isolation occurred despite the fact that the individual was living in a family or couple relationship:

*I consider myself very different to other people growing up… going to school and getting picked on for being different and for being really small, like I was tiny, I don’t even believe how small I was. I guess, it sort of painted an image for what I thought people were like…That feeling of emptiness and that real, pure loneliness feeling of ‘it’s just me in the world’. And even though I was in the city and there was all these people, just thinking I was so alone*—Male, 1 week in treatment for amphetamines.

*I started to feel more and more depressed and more excluded from the outside world. I didn’t enjoy it very much…I suppose my worst point was about 6–8 months ago. I lost my dog, and that was pretty much the only thing connecting me to the rest of the world at that time*—Male, 4 weeks in treatment for alcohol.

For many of these participants, substance use was associated, at least initially, with membership of a new social group and an associated gain in social identity. Some people described how substance using networks offered a sense of belonging and acceptance that had been absent previously:

*All I cared about was fitting in with some people and I found that through bad kids and gangs, and sort of the crime, and all that kind of lifted. Obviously drinking, my older sister introduced me to drinking when I was 12, by the age of 13 I was pretty much binge drinking every day at school.*—Male, 1 week in treatment for amphetamines.

*I hung around the wrong people. I learnt by being naughty I could have friends that actually liked me, that wanted to be with me. Well not be with me, be around me—*Male, 1 week in treatment for amphetamine misuse.

### Relationships as Triggers for Entering Treatment

Even for those participants whose substance use initially offered a sense of belonging and a positive identity, this shifted as their substance use became more extreme and maladaptive over time, to the point where some experienced rejection from their using groups. This was reflected in conversations or confrontations with others when the participant was using the “wrong” substances, at the “wrong” times, or in the “wrong” quantities. This prompted entry into treatment for some participants, despite some ambivalence about giving up their substance use.

*They have alcohol problems, but they don’t think that they do. They say ‘why don’t you just sit down and have a beer’, ‘be a normal person’…‘Why do you have to take this valium crap?’*—Female, 9 weeks in treatment for opiate and benzodiazepine misuse.

*My mate who I lived with used to smoke pot and drink but he never used to drink as much. He told me that I was getting worse and worse, and eventually he said “in my consensus I think you are an alcoholic now”*—Male, 4 weeks in treatment for alcohol misuse.

There were some expressions of fear or uncertainty about entering treatment and what it would be like:

*I was scared when I heard there were people here from jail but I can tell you I have met some of nicest gentlemen in my life that have come from jail. I am truly fond of them! So this place has taught me to like men. I thought all men were bad, but now I’m living with them, I love them all dearly*—Female, 6 weeks in treatment for polysubstance use.

It was common for participants to cite an important person or social group as a trigger for entering treatment and turning their lives around. For some, this motivation came from conversations with a member of their family:

*I sought out my Aunty, she detoxed me for a month. She showed me the quality of life I could be living. The importance of family, and I really got that back.—*Male, 2 weeks in treatment for heroin.

In some cases, this pivotal help came from a professional:

*He was … giving me the time of day and no one else would, he was the first person in 6 months who stopped and said hello. Everyone else would just look at me and keep walking. I said to him, so what do you do? He goes, I’m the local paramedic in town….*—Male, 3 weeks in treatment for cannabis misuse.

For others this turning point involved a realization that they’d lost their whole social network:

*It was um my birthday and ….there was nobody there. I was just on my own and I’m a very spiritual believer and I felt that I had a sign and my angels tell me “have a look at yourself – there’s nobody even here and it’s your birthday”. The phone wasn’t ringing, Nothing. I just grabbed the local directory and it opened on the page with [therapeutic community] on it*—Female, 5 weeks in treatment for opiates.

### Development of a Recovery Identity

Most participants described positive ties with other residents and staff of the TC and a sense of belonging and acceptance within the community. Being a member of the TC allowed the development of positive social connections and, for some clients, a positive identity:

*…the support was just instant straight away from everybody. Everybody. Nobody looked at you sideways they were just continually there. And within a few days I was just stoked that I was here and I had no inclination to leave at all. I still don’t…. To have these people around is just amazing. When people leave it’s just like “Nooo!” when they haven’t finished – especially when they’re young and they’ve got so much to gain out of it and don’t realise it yet*—Female, 5 weeks in treatment for opiate misuse.

*It’s like building the foundation of a house, and all the community and staff have had input into building that foundation for me. The foundation isn’t ready yet and has to be rooted in firm strong ground otherwise I’ll just sink in the sand. I want to be strong and I don’t want to quit*—Female, 6 weeks in treatment for poly-substance misuse.

Participants reported that they were able to receive emotional support and understanding from fellow members of the community who had experienced similar life circumstances and there is evidence from some of these comments that clients formed a single coherent entity within the community:

*Although, everyone was just so supportive; sometimes it felt like they are psychic because I would just be sitting somewhere having a cigarette and someone would ask “How you travelling in your head?” and I would think, “How did you know I was in that bad head space right now? I have just been talking and smiling with you a few minutes before. I was just having a cigarette and didn’t think I had an odd look on my face”*—Female, 3 weeks in treatment for alcohol misuse.

*Yeah, and being in a community; I’m just not really used to that you know? Your drug circle is pretty limited, [but here] you get to know a lot of people who all have similar sorts of issues*—Male, 4 weeks in treatment for poly-substances.

Some of the participants made a special mention of their relationship with a counselor as important in their recovery:

*You couldn’t have anything more healing than being able to talk to someone who knows exactly what you’re talking about. From beginning to end… your losses, your gains… everything*.—Female, 5 weeks in treatment for opiate misuse.

Participants also described the TC as a place where they had a sense of purpose, and a meaningful and important role within the community:

*You have to work your way into these positions. You become a mentor – leading a new person into life at [the therapeutic community]. Took a while for me to get to be a mentor. I was stubborn – everything was black and white – so it took a while for me to be settled in [the therapeutic community] in a way that I could mentor a new person. The next step is residential house manager. You are in control of 10–12 people in the house*—Male, 27 weeks in treatment for alcohol misuse.

Despite the widespread experience of social cohesion and support within the TC, a minority of participants found it difficult to integrate with the community and there was some ambivalence expressed about this process:

*[I’ve been]…noticing a fair bit of passive aggressive stuff. For example, people saying things to other residents like “I have been noticing you have been getting quite a lot of consequences lately. Is there anything we can do to support you, anything I can do to help?” I look at the person and think, “you’re not actually wanting to support them. You are pointing out to them their consequences and relishing in their failures”. So I’m really just getting to know the deeper levels of the personalities…. But it is not as solid of a community as I thought it was. You know, everyone is from different walks of life and different addictions they are trying to recover from, you can’t expect…you know…[a perfect community].*—Female, 3 weeks in treatment for alcohol misuse.

Some participants articulated a view that the social connections formed within the TC were temporary and that they were unlikely to continue these relationships after their treatment.

*Um…not particularly [I don’t have close relationships within the TC]. No. I guess working in the field with aged people you have got to have a sense of detachment, because knowing they are gonna pass on. I guess I brought a little bit of that into me. They are going to go their own way, know what I mean? This is my theatre production. And you know, I choose who I want to reveal stuff to…there would be very few people at this point who I would stay connected with after I leave.*—Male, 4 weeks in treatment for alcohol.

For some participants, it was too early in treatment for them to comment on their future plans. However, many were making clear plans of who they wanted to be and how they wanted to live after leaving treatment. Here we see the re-emergence of the two pathways, with the participants who previously held positive social identities hoping to renew these, and those who lacked social connections and identities aspiring to develop new positive identities and social roles.

### First Pathway After Treatment—Renewed Identities

Some participants hoped to renew and repair positive social identities that they had held prior to the addiction such as occupational and educational identities:

*To be able to achieve my dreams, and that is to be a professional fighter. That is one of the things that I want to be able to do. I trained for 9 years doing it, and I really want to be able to do that*—Male, 4 weeks in treatment for alcohol misuse.

*I really value education. I need to [finish] my education, I need to get somewhere good. Coz I don’t want to be a bum the rest of my life, I’m better than that.*—Female, 26 weeks in treatment for poly-substance use.

### Second Pathway After Treatment—Aspirational Identities

For many, their goals were closely aligned with new aspirational identities such as “university student” or “writer”:

*For me, I have, the good side of me, I want to go to Uni, I want to do occupational therapy and I want to be able to have a normal life. That’s my real ambition. And to give back to society, coz I know I’ve taken a lot from the society and I really want to give back. I really want to educate troubled children or youth and inspire them*—Male, 1 week in treatment for amphetamine use.

*I think all of my self-esteem was just crushed that I had none left, but slowly bit by bit it is coming back. But I want a lot more. Now I am aspiring to write a book.*—Female, 6 weeks in treatment for poly-substance use.

For others, their aspirational identity was associated with family roles such as “spouse,” “parent,” or “grandparent”:

*I want kids, I want a family, I want to get married and I want to see my Mum’s face when she sees my first kid. My sister just had her first kid, the day before I went into detox, which makes me an uncle. Which is the first time in my life that I have had a responsibility like that, and I am so happy that I am an uncle because there is nothing better than children I believe, because innocence is one of the best things in this world*—Male, 4 weeks in treatment for alcohol.

In contrast to the participants who indicated that their social connections with others in the TC would be transient, others intended to maintain their recovery identity through continued contact with the TC:

*Yeah so it’s mainly the tools and I won’t ever leave here in that way that I’ll be – that’s why [previous treatment center] didn’t work, because I left too early and I didn’t have any backup like we can keep coming here for rap groups, after care, you can come here for lunch, you can come here for the morning meeting.*—Female, 5 weeks in treatment for opiates.

## Discussion

The thematic analysis of interviews with 21 adults undertaking treatment in a drug and alcohol TC revealed that people who have experienced addiction understand their own substance misuse and recovery through their relationships and their social identities. Participants clearly articulated how they saw themselves and their circumstances before the development of an addiction in relation to their social roles and relationships. Participants communicated how their identities shaped—and were shaped by—the development of an addiction. They described both losses and gains of social networks as they entered treatment in the TC. Finally, participants imagined their future in terms of new or renewed social roles and relationships.

### An Identity Loss Pathway

In terms of the addiction literature, this investigation is the first to suggest two alternative pathways into addiction—one in which addiction represents an *identity loss* and one in which addiction brings with it an *identity gain*. In the current study, people spoke about a number of sources of positive pre-addiction social identities: abilities, family roles, work roles and relationships. The development of addiction impacted negatively on participants’ ability to maintain these identities—for example, the young man whose budding sporting career was cut short by the misuse of drugs. Addiction also impacted on participants’ behavior in ways that caused damage to their relationships—for example, the man who deceived his family and colleagues by continuing to get dressed for work each day but then returned home to drink instead. The negative impact of identity loss has been found in other research on people going through life transitions—such as, loss of identities when one suffers a stroke (Haslam et al., 2008); loss of home and family ties when students go away to university (Iyer et al., 2009).

For participants following the identity loss pathway, their substance user identity was described as a negative, devalued identity: being a “junkie” or “dealer.” Several participants felt that substance use had led them to feel stigmatized and the criminal or devious behavior associated with obtaining and using the substance had “spoiled,” or tainted, their identity. This resonates with previous research by Waldorf (1983), Waldorf and Biernacki (1981), Hughes (2007), McIntosh and McKeganey (2000), and the “AA story” that came out of Hanninen and Koski-Jannes’s, (1999) narrative analysis. As these earlier researchers noted, participants’ desire to repair their spoiled identity appeared to create the motivation for ceasing substance use and its associated way of life. Similarly, Hill and Leeming’s, (2014) interview-based study of six adults recovering from alcohol dependence found that the participants were very much aware of stigmatized identity that was assigned to them as “alcoholics.” However, to some extent these participants were able to avoid stigma by viewing themselves in terms of an “aware alcoholic self” which was different from their previously unaware self and formed the basis for a new and valued identity.

Socially meaningful others often motivated people’s decision to enter treatment, either directly through discussion—such as the young man who turned to his Aunt for detoxification and support—or indirectly as an attempt to salvage a lost or damaged relationship—such as several participants who spoke about wanting to repair their relationship with their children, or be present for their grandchildren.

### An Identity Gain Pathway

A lesser known second pathway was apparent among those who were socially isolated or who lacked supportive social ties or who had unmet identity needs. These participants appeared to be drawn to the user identity due to its promise of belonging and esteem, making them vulnerable to normative peer influence (Newcomb and Bentler, 1989)—such as those who started taking drugs with older relatives or fell in with the “wrong crowd.” These participants described a sense of belonging in the substance using (and sometimes drug dealing) social networks that brought with it new esteem and social support and even, in one participants’ case, a sense of power over others.

Although details of their upbringing were beyond the scope of the study, some participants alluded to family dysfunction or abuse and the onset of addiction as a means of rebellion or escape from their family. This theme was consistent with Ary et al. (1999) findings that substance use and other problematic behaviors among young adults were predicted by social factors such as poor parental monitoring and associations with deviant peers. For participants on the identity gain pathway, substance using groups were often their first experience of acceptance and community. However, as their substance use became more extreme and maladaptive over time, they often experienced rejection from their using groups—in some cases, for using the “wrong” substances, or for losing control over their substance use. This was an alternative way in which socially valued networks prompted entry into treatment for some participants, albeit with a somewhat ambivalent desire for recovery.

### Joining the Therapeutic Community

Although many participants expressed a fear or uncertainty about entering treatment, once in the TC, it was common for participants to report a sense of group belonging and cohesion (Dingle et al., 2015). There was evidence of mediators mentioned in previous research on mutual support groups and other group treatments for addiction such as collective esteem, normative structure around abstinence, and social support and control (Moos, 2007; Groh et al., 2008; Frings and Albery, 2015). It has also been found in psychotherapy groups that outcomes are related to the extent to which these groups foster a perception of normative change (Cruwys et al., 2015). During treatment in the TC, participants clearly gave and were able to accept support from other members of the community and from counselors (Crabtree et al., 2010). That members of the TC were supportive of one another is consistent with earlier findings that social identity is the basis for giving and receiving social support (Haslam et al., 2005). However, on occasion, the motives behind such support and the subsequent responses they invoked were not perceived as entirely positive.

It is important to note that these participants were not simply passive recipients of treatment—rather they took an active role in their own therapeutic process as well as work roles within the TC, in the gardening, catering or housekeeping teams, and some taking on peer mentor status and then becoming a house manager or community leader. In this way, the social roles within the TC may have informed and strengthened the participants’ recovery identity, such as the young man who spoke about working his way into a mentor role in which he inducted new people into life at the TC. In line with the concept of an identity gain pathway, the taking on of active roles within the TC could be seen as a form of identity performance; that is, the public enacting of identity relevant norms that serve to consolidate the group identity, an expression of the message: “look at me living a sober, healthy life and serving my TC” (Klein et al., 2007). These active roles contrast with other services in which clients are passive recipients of help (e.g., hospital and primary care services and some homeless accommodation services). The opportunity to take an active role appears to be an important factor in treatment outcome according to research showing that autonomous individuals achieve positive outcomes from therapeutic and behavior change programs (Dwyer et al., 2011; Ryan et al., 2011). This is also consistent with the helper therapy principle (Moos, 2007; Pagano et al., 2011) that demonstrates how becoming a buddy or helper within AA group is associated with positive long term abstinence from alcohol.

### After Treatment—Recovery Identity Continuity and Renewed or Aspirational Identities

Although not all participants envisaged an ongoing connection with others in the TC, many did, through living at the halfway house and coming back to the TC for support group sessions and graduations of their peers. Regardless of whether they were planning to engage in ongoing contact with the TC, many participants indicated that their social identity as a member of a “recovery” social network would continue after their treatment. In addition to this, the participants who described positive pre-addiction identities spoke about renewing these former identities—for example, the man who wanted to go back to training to be a professional fighter, and the woman who wanted to finish her education. In contrast, the participants who started with a relatively impoverished social network tended to aspire toward new social identities—for example, the young man who dreamed of becoming a husband and father.

### Clinical Implications

These emerging themes raise questions about how a person’s social identity history might influence treatment outcomes and whether different approaches to treatment might be indicated. For instance, should interventions be aimed at renewing or repairing “spoiled identities,” or in fostering new identities for socially isolated individuals—such as is implied in the lyrics of the song “Rehab” at the start of this paper? One possibility is that such treatment decisions should be based in part on whether an identity “loss” or “gain” pathway is most reflective of an individual’s experience of addiction up to that point. Under what circumstances do a person’s social networks and ties facilitate recovery or relapse? It is likely that a single approach will not fit all, and this suggests a need for routine assessment of social roles, networks and identities early in treatment to understand the “push” or “pull” of the addiction identity as part of the treatment process. This could be achieved via a social mapping activity (Best et al., 2014; Haslam et al., 2016), conducted early in the person’s treatment, combined with motivational interviewing procedures using the person’s positive identities and social roles as motivating factors for change (Miller and Rollnick, 2002).

The sometimes transient nature of identities related to TCs and recovery raises an interesting question of how long these identities persist beyond the controlled environment of a TC, how they are maintained (or not) over time and if they remain beneficial. Such transitional identities could be functional in helping the individual transition from the TC back into society but then become unnecessary; acting as “disposable” identities whose use is constricted to periods where they have utility. This echoes the notion of “disposable ties” found in the urban poor who rely on strangers rather than family or established social network to meet their immediate needs (Desmond, 2012). Existing literature (e.g., Kelly et al., 2011; Litt et al., 2015) highlights the significantly lower relapse risk for those who have a strong recovery support network in place. The maintenance of recovery-oriented identities forged in TCs may foster engagements in such networks beyond treatment. This is an area for future research but, if it is the case, adds further emphasis to the need for clinicians to explore identity-based interventions as part of the treatment process. Halfway houses and follow-up groups would be one way in which TC identities could, where appropriate, be reinforced. In addition to treatment-specific groups, clinicians could assist clients to join interest-based community groups toward the end of their treatment that they can continue to belong to after treatment as a way of “bridging” their social networks and supporting their ongoing recovery. These groups need not revolve around addiction recovery—research with choirs, art groups and sports/exercise groups provide evidence that this approach shows benefit for both substance use and mental health more generally (Dingle et al., 2013, 2014; Cruwys et al., 2014b).

### Theoretical Contributions

From a social identity perspective, this is one of the first attempts to track the experience of identity change over time and using a detailed qualitative analysis of participants’ experiences. Although social identity has been linked with a huge number of health behaviors in cross-sectional and experimental contexts, few studies have examined identity transitions and their health implications longitudinally (with some few exceptions, e.g., Iyer et al., 2009; Cruwys et al., 2014b; Dingle et al., 2015). The results of the current study suggest that the dynamics of identity change across months and years shape a person’s life in fundamental ways—addiction is not only something that people experience as an individual biological reality (as it has often been studied), but rather as a psychosocial phenomenon which in part reflects individuals’ attempts to navigate their social world. The thematic analysis has yielded a theoretical model that lends itself to further quantitative testing in a range of other samples and treatment settings.

### Limitations/Questions Raised for Future Work

Social identities are likely to be culturally bound—the stigmatization of addiction, the development of “disease” models, and differing understandings and definitions of addiction may mean that the form and content of identities vary across cultures and over time. The relationship between identities may also differ as a function of their content (see, e.g., Buckingham et al., 2013). Despite this limitation, the current research speaks to basic identity processes that are likely to be generalizable to the extent social identity principles seem to be robust across cultures (Suh, 2002). One further limitation of the current study is in the retrospective nature of the measures taken. It could be argued that part of the process of recovery from SUDs is reflecting on (and imposing meaning upon) past life events. Thus, it is possible that the meaning of events and *post hoc* construction of identities (i.e., being a “junkie”) reflect a *post hoc* justification for (or understanding of) past events. Such a construction could be a product of the treatment process, rather than an in-the-moment reflection of identity. Whilst this is possible, we also note that many of our interviewees reflected on their present and future identities (including many currently affiliating with addiction related identities), as well as those from the past. Further, other more quantitative work has supported the predictive nature of identity prospectively (e.g., Dingle et al., 2015).

Finally, the current study did not explore directly how repeated treatment episodes affect identity. One possibility is that a cycle of relapse-treatment-relapse may make addiction identities seem more intractable whilst simultaneously making identities associated with recovery less resilient and, perhaps, achievable. In contrast, this same pattern may well alter the content of the “addict” identity (as involving relapse and being constantly present) and also that of recovery (e.g., to include ideas of persistence in the face of adversity) in way which foster recovery. Although such analysis is beyond the scope of the present paper, it does raise interesting questions as to how clinicians discuss identity and relapse to best effect.

## Conclusion

In summary, the current study found two social identity-related pathways into and out of drug and alcohol addiction. For some, the development of the addiction was associated with a loss of positive social identities and these people were motivated to enter treatment in part to restore their former social identities and roles. Others were socially isolated and the addiction represented an identity gain. For this latter group, treatment required a giving up of the addiction social networks and an aspiration to new positive social identities and roles. Membership of the TC itself was experienced by most participants as a valued identity but the expectation that this recovery identity would continue beyond treatment varied among participants. In summary, both substance use related and other social identities could be assessed and addressed during treatment as an important motivational force for change and a guide for the kind of social changes that might be most helpful to the individual in supporting his or her ongoing recovery from addiction.

### Conflict of Interest Statement

The authors declare that the research was conducted in the absence of any commercial or financial relationships that could be construed as a potential conflict of interest.
